# Quantitative hemodynamic imaging: a method to correct the effects of optical properties on laser speckle imaging

**DOI:** 10.1117/1.NPh.10.4.045001

**Published:** 2023-10-03

**Authors:** Thinh Phan, Christian Crouzet, Gordon T. Kennedy, Anthony J. Durkin, Bernard Choi

**Affiliations:** a University of California, Irvine, Beckman Laser Institute and Medical Clinic, Irvine, California, United States; b University of California, Irvine, Department of Biomedical Engineering, Irvine, California, United States; c University of California, Irvine, Department of Surgery, Irvine, California, United States; d University of California, Irvine, Edwards Lifesciences Cardiovascular Innovation Research Center, Irvine, California, United States

**Keywords:** cerebral hemodynamics, laser speckle imaging, spatial frequency domain imaging, tissue optics, diffuse optics

## Abstract

**Significance:**

Studying cerebral hemodynamics may provide diagnostic information on neurological conditions. Wide-field imaging techniques, such as laser speckle imaging (LSI) and optical intrinsic signal imaging, are commonly used to study cerebral hemodynamics. However, they often do not account appropriately for the optical properties of the brain that can vary among subjects and even during a single measurement. Here, we describe the combination of LSI and spatial-frequency domain imaging (SFDI) into a wide-field quantitative hemodynamic imaging (QHI) system that can correct the effects of optical properties on LSI measurements to achieve a quantitative measurement of cerebral blood flow (CBF).

**Aim:**

We describe the design, fabrication, and testing of QHI.

**Approach:**

The QHI hardware combines LSI and SFDI with spatial and temporal synchronization. We characterized system sensitivity, accuracy, and precision with tissue-mimicking phantoms. With SFDI optical property measurements, we describe a method derived from dynamic light scattering to obtain absolute CBF values from LSI and SFDI measurements. We illustrate the potential benefits of absolute CBF measurements in resting-state and dynamic experiments.

**Results:**

QHI achieved a 50-Hz raw acquisition frame rate with a 10×10  mm field of view and flow sensitivity up to ∼4  mm/s. The extracted SFDI optical properties agreed well with a commercial system ($R^{2}\geq 0.98$). The system showed high stability with low coefficients of variations over multiple sessions within the same day (<1%) and over multiple days (<4%). When optical properties were considered, the *in-vivo* hypercapnia gas challenge showed a slight difference in CBF (−1.5% to 0.5% difference). The *in-vivo* resting-state experiment showed a change in CBF ranking for nine out of 13 animals when the correction method was applied to LSI CBF measurements.

**Conclusions:**

We developed a wide-field QHI system to account for the confounding effects of optical properties on CBF LSI measurements using the information obtained from SFDI.

## 1 Introduction

The brain is a nutrient-demanding organ whose hemodynamics are well regulated to ensure proper functional activities. Blood flow in the brain is tightly controlled to maintain homeostasis (i.e., vascular reactivity) and to deliver sufficient glucose, oxygen, and other nutrients to support neuronal activities (i.e., neurovascular coupling). The neurovascular unit plays a crucial role in controlling essential vascular activities, such as vasodilation and constriction to regulate cerebral hemodynamics.^1^^,^^2^ Studying cerebral hemodynamics at resting states or in relationship to perturbations may provide diagnostic or prognostic information on the onset or progression of brain conditions, such as stroke and Alzheimer’s disease (AD).^1^^,^^3^^,^^4^

In preclinical models, the change in cortical hemodynamics can be quantified using reflectance techniques, such as laser speckle imaging (LSI) and optical intrinsic signal imaging (OISI).^5^^–^^8^ LSI measures cerebral blood flow (CBF),^9^ while OISI measures hemoglobin concentrations and their derivative parameters, such as cerebral blood volume and tissue oxygen saturation (${\mathrm{StO}}_{2}$).^5^^,^^10^^–^^12^ With LSI, local speckle contrast values approximate the speckle decorrelation time, which is assumed to be inversely proportional to the speed of moving scatterers, such as red blood cells.^9^ With OISI, spectroscopic analysis can be applied to the measured reflectance changes at multiple wavelengths to obtain chromophore concentrations such as deoxyhemoglobin (HbR) and oxyhemoglobin (${\mathrm{HbO}}_{2}$).^10^^,^^11^ LSI and OISI have been used in tandem to obtain a more complete view of hemodynamics following stimulation and in disease models.^6^^,^^13^^,^^14^

Optical scattering complicates the interpretation of OISI and LSI data. Measured differences in reflectance are affected by the absorption and scattering properties of the interrogated tissue. Scattering lengthens the path length over which the detected reflected light travels in an unknown manner. With planar reflectance measurements alone, it is challenging to separate the effects of scattering from absorption. Hence, OISI measurements oftentimes are normalized to the corresponding initial values, to estimate relative changes in HbR and ${\mathrm{HbO}}_{2}$ during time-resolved reflectance measurements while assuming scattering remains constant. With LSI, speckle contrast values are affected by changes in optical properties.^15^ Specifically, an increase in absorption can increase the measured speckle contrast despite a constant flow value. The opposite applies to scattering, where an increase in scattering results in a decrease in speckle contrast.^15^^–^^17^ Nonetheless, this dependency of speckle contrast on optical properties is not typically considered in studies that employ LSI.

Spatial frequency domain imaging (SFDI) enables the quantitative measurement of wide-field spatially resolved optical properties.^18^^,^^19^ Using a spatially patterned illumination, in combination with an appropriate model of light propagation in turbid media, SFDI can determine pixel-wise mapping of absorption ($\mu_{a}$) and reduced scattering (${\mu_{s}}^{\prime }$) coefficients. Within the context of rodent neuroimaging, SFDI was previously used to study the difference in brain optical properties of wild-type and AD mouse models.^20^^,^^21^ We also reported on a combination of LSI and SFDI in a fast imaging system to image cortical hemodynamics in a rat model of cardiac arrest.^16^^,^^22^^–^^24^ However, in these investigations, the LSI and SFDI fields of view (FOV) were mismatched in terms of size, temporal synchronization, and spatial overlap and thus required additional post-acquisition image alignment and analysis. Furthermore, the LSI and SFDI data were used strictly in a complementary manner, and tissue’s absorption and scattering effects on LSI data were not addressed.^16^

Here, we introduce a quantitative hemodynamic imaging (QHI) system that combines LSI and SFDI into an integrated device, with matching FOV and temporal synchronization. Details on hardware setup and software for acquisition and analysis are provided. We provide system characterization results for sensitivity, accuracy, and precision tests using tissue-mimicking silicone phantoms. Using the direct measurement of optical properties from SFDI, we describe a method derived from the dynamic light scattering literature to obtain an absolute CBF value from LSI and SFDI measurements. Finally, we illustrate the potential benefits of such absolute measurements in resting-state and hypercapnia gas challenge mouse models.

## 2 System Instrumentation

### 2.1 SFDI Hardware

**Fig. 1:**
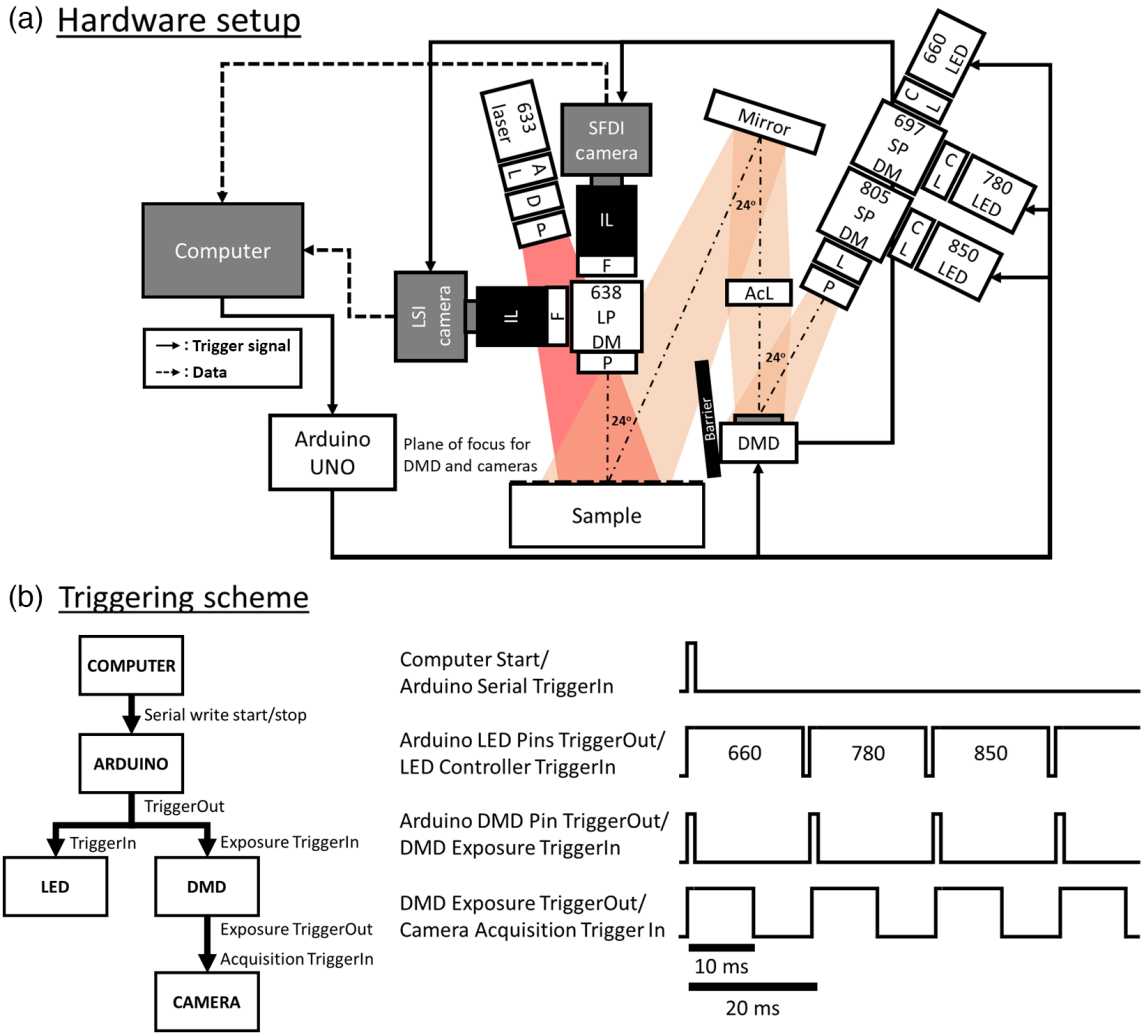
Overall schematic of the QHI system. (a) Hardware setup of QHI with LSI and SFDI components [AL, aspheric lens; AcL, achromatic lens; CL, condenser lens; D, diffuser; DMD, digital micromirror device; F, appropriate notch (for SFDI) and line filters (for LSI) at LSI wavelength, IL, imaging lens with spacers; L, plano-convex lens; LED, light emitting diode; LPDM, long-pass dichroic mirror; P, linear polarizer (cross-polarized between illumination and detection arms); and SPDM, short-pass dichroic mirror]. (b) Triggering scheme of the system where the computer sends a serial trigger to activate an automatic Arduino triggering scheme for the LED and the DMD. The electrical output of the DMD was then used to trigger the camera. The exposure time was set to 10 ms with a 20 ms period per full cycle, allowing for an effective 50 Hz raw frame rate. The Arduino triggering scheme can be deactivated with another serial trigger from the computer.

The hardware design and assembly of the SFDI arm of the system were based on the openSFDI guide^25^ by Applegate et al.,^26^ with adjustments to enable continuous acquisition. Since the typical mouse heart rate ranges from 5 to 14 Hz, we designed QHI to achieve raw acquisition frame rate of 50 Hz to capture pulsatile signals using LSI. This frame rate allowed for a slower effective SFDI frame rate of 2.8 Hz for three wavelengths and two spatial frequencies. On the detection arm, a fast CMOS camera (BFS-U3-32S4M-C, FLIR, Oregon, United States) was mounted so that the imaging axis was orthogonal to the sample plane [Fig. 1(a)]. We used a 50 mm fixed focal length lens (67-717, Edmund Optics, New Jersey, United States) at maximum zoom together with a 10 mm spacer (CML10, Thorlabs, New Jersey, United States) to achieve a ∼10×10  mm FOV (magnification = 0.42) at 1200×1200  pixel resolution. The FOV was chosen to match the dimensions of a mouse cranial window. The working distance was ∼130  mm. A notch filter at 632.8 nm (86-126, Edmund Optics, New Jersey, United States) was used to minimize possible crosstalk with the LSI wavelength [Fig. 1(a)].

The SFDI illumination arm was configured with an illumination axis at ∼24  deg with respect to the imaging arm [Fig. 1(a)] to minimize distortion artifacts (e.g., keystone artifacts) on projected SFDI patterns within the FOV. All optical components were set up at 190 mm away from the mounting breadboard to provide ample space for animal imaging. The projection system included three LEDs at 660, 780, and 850 nm (M660L4, M780LP1, and M850L3, respectively; Thorlabs, New Jersey, United States) coupled into a digital micro-mirror device (DMD) projector (DLP4500 NIR, Keynote Photonics, Texas, United States) using appropriate dichroic mirrors (FF697-SDi01-25x36, Semrock, New York, United States, and DMSP805R Thorlabs, New Jersey, United States) and condenser lenses (ACL2520U-B, Thorlabs, New Jersey, United States) [Fig. 1(a)]. The three wavelengths were chosen to enhance sensitivity to the chromophores of interest (i.e., HbR and ${\mathrm{HbO}}_{2}$) based on their absorption spectrum and to approximately match the probing depth of the LSI wavelength (633 nm). To achieve sufficient light intensity for fast acquisition (i.e., 50 Hz), we added a plano-convex lens (LA1708-AB, Thorlabs, New Jersey, United States) in front of the combined light beam before the DMD [Fig. 1(a)]. The plano-convex lens position was carefully adjusted to avoid creating a focused image of the LED on the DMD. A 400 to 1100 nm achromatic lens (AC254-075-AB-ML, Thorlabs, New Jersey, United States) was positioned ∼100  mm from the DMD so that a focused image of roughly 3× magnification of the DMD’s display was formed on the focal plane. We then made fine adjustments to ensure that the illumination and detection arms were confocal using projection alignment steps listed in the openSFDI guide.^27^ A barrier was then added to block unwanted diffraction patterns from the DMD from the imaging axis. We controlled the LEDs using dedicated drivers (LEDD1B, Thorlabs, New Jersey, United States) with triggering signals from an Arduino Uno (1050-1024-ND, Digi-Key Electronics, Minnesota, United States). Details regarding the electrical configuration are discussed in the Acquisition Software section below.

Finally, linear polarizers (LPNIRE100-B, Thorlabs, New Jersey, United States) were added to achieve crossed polarization between the illumination and detection arms while maintaining appropriate intensity counts (i.e., 40000 to 45000 counts for 16-bit images) from a static silicone phantom of known optical properties ($\mu_{a}=0.019$, 0.019, and $0.019\;{\mathrm{mm}}^{-1}$, ${\mu_{s}}^{\prime }=1.07$, 0.83, and $0.72\;{\mathrm{mm}}^{-1}$ at 660, 780, and 850 nm, respectively).

### 2.2 LSI Hardware

For the LSI detection arm, an identical CMOS camera and lens-spacer system to that of the SFDI arm were used to achieve a matching FOV. We combined the two detection arms using a 638 nm long pass dichroic mirror (DMLP638R, Thorlabs, New Jersey, United States) [Fig. 1(a)]. For LSI, we adjusted the imaging lens aperture to a f/# of 4 to achieve a proper speckle-to-camera pixel size ratio (approximately 9  μm to 3.45  μm, respectively).^28^ A 632.8 nm laser line filter (FL632.8-3, Thorlabs, New Jersey, United States) was used to minimize crosstalk with illumination from the SFDI arm [Fig. 1(a)].

For the LSI illumination arm, a long-coherence 633 nm laser (115-81059-554, Coherent, California, United States) was coupled with an aspheric lens (C330TMD-B, Thorlabs, New Jersey, United States) to quickly expand the beam. A 1500-grit diffuser (DG-10-1500-MD, Thorlabs, New Jersey, United States) was used to homogenize the beam. The laser was then mounted to achieve homogeneous illumination over the camera’s FOV with the beam concentric with the FOV [Fig. 1(a)]. We adjusted the laser’s illumination power using a polarizer (LPNIRE100-B, Thorlabs, New Jersey, United States) to achieve the appropriate intensity count (i.e., 60-70 for 8-bit images) on the same static silicone phantom used for the SFDI setup. Similar to the SFDI arm, cross-polarization with linear polarizers was used to minimize specular reflectance [Fig. 1(a)].

### 2.3 Computer Specification and Electrical Wiring

The performance and effective frame rate of the system also depend on the specifications of the acquisition computer. Here, we used a laptop with Intel^®^ Core™ i7-9750H CPU (12 CPUs) processor, 16 GB RAM, and a 1TB built-in M.2. solid state drive (SSD). The LED drivers were connected to the Arduino triggering digital pins using BNC cables with the ground leads connected to the Arduino GND pin. On the DMD, the “TRIG IN” connector J7 was connected to the Arduino DMD trigger pin and the “TRIG OUT” connector J11 to the cameras’ trigger-in pin through GPIO cables. All ground outputs from the DMD and the cameras were connected to the Arduino GND pin. Micro-B-to-A-male USB-3.0 cables were used to connect the cameras and the computer. One B-to-A male USB-2.0 cable was used to connect the Arduino to the computer.

## 3 Acquisition Software

We developed a master acquisition program in LabVIEW (LabVIEW 2020, NI, Texas, United States) to control the Arduino and camera acquisition. For camera acquisition, we used the NI Vision Acquisition Software suite to set the camera’s acquisition mode to external triggering, gain to zero, and exposure time to 10 ms. Gamma correction was disabled, and trigger delay time was set to 11  μs (i.e., smallest value). The image format was set to uncompressed TIFF at 1200×1200  pixel resolution with a bit depth of either 8-bit (LSI) or 16-bit (SFDI), aligned to the center of the sensor. We used the LabVIEW master program to write a serial trigger for the Arduino to activate an autonomous triggering scheme [Fig. 1(b)]. Here, the autonomous triggering scheme stops after the Arduino receives a deactivation serial trigger from the computer.

The autonomous Arduino triggering scheme includes cycling through wavelengths first and then spatial patterns to mitigate temperature-dependent drifting of LED intensity. The code controlled the timing of the DMD and the LED. Here, we ran the DMD in pattern sequence mode with an external trigger (refer to Keynote Flexlight LC4500 LC4500-RGB-EKT Electronics Kit User’s Guide Sec. 3.3.2^29^). Images of the chosen spatial frequencies ($f_{x}=0$ and $0.3\;{\mathrm{mm}}^{-1}$) at three phases (0 deg, 120 deg, and 240 deg) were loaded onto the DMD’s internal memory. We note that the DMD’s maximum speed was limited by the loading time of the images. Here, only two 24-bit images could be loaded and displayed at a full frame rate.

Next, an input signal from the Arduino switched the LEDs for every frame and advanced the pattern once every three frames via TTL triggering. The exposure and period time of the DMD were set to match the camera’s exposure time of 10 ms. The trigger output signal from the DMD was delivered 11  μs before the pattern display to account for the camera’s 11  μs minimum trigger-to-exposure delay time. The delay time between two consecutive triggering cycles was set to 20 ms, which effectively resulted in a raw 50 Hz raw acquisition rate for LSI and SFDI [Fig. 1(b)]. For our current computer specifications, this frame rate was appropriate to minimize frame-dropping issues. This led to effective 2.8 and 50 Hz frame rates for SFDI and LSI, respectively.

The images were saved with time stamps and an order index of 0 to 17 (a total of 18 frames needed for SFDI at 3 wavelengths, 2 spatial frequencies, and 3 phases). The indices were later used to check and account for any dropped frames during analysis.

## 4 Processing Software

Due to fast acquisition at full uncompressed 1200×1200  pixel resolution at 8- and 16-bit depth (i.e., 216 megabytes per second) on a single SSD hard drive, we expected incidents of dropped frames. We noted that dropped frames happened when there were background tasks such as file transferring and Windows updates. Thus, we used the indices of each successfully saved image to check for a complete set of 18 consecutive frames in LSI and SFDI data (i.e., consecutive indices from 0 to 17) before performing further processing steps listed below. If a dropped image was detected in either LSI or SFDI, the entire set of 18 frames for both modalities was disregarded in further processing steps.

### 4.1 SFDI Processing Pipeline

We used MATLAB (R2019b, MathWorks, Massachusetts, United States) to process SFDI data following a previously published protocol.^19^ The first step of the processing scheme was pixel-wise spatial frequency demodulation using the three phases collected to obtain the AC signal of the reflectance

$$M_{\mathrm{AC}}(f_{x})=\frac{\sqrt{2}}{3}{\left[(I_{1}-I_{2}{)}^{2}+(I_{2}-I_{3}{)}^{2}+(I_{3}-I_{1}{)}^{2}\right]}^{\frac{1}{2}},$$ (1)

where $I_{1}$, $I_{2}$, and $I_{3}$ are reflectance images at a spatial frequency $f_{x}$ at 0 deg, 120 deg, and 240 deg phases, respectively. A Gaussian filter (15×15  pixel window size) was applied to the demodulated AC signal images to remove noise. The AC images ($M_{\mathrm{AC}}$) were then calibrated against the calibration phantom $M_{\mathrm{AC},\text{phantom}}$ and a diffuse reflectance value of the phantom ($R_{d,\text{phantom}}$) obtained from a white Monte Carlo (WMC)^30^ simulation to obtain a diffuse reflectance ($R_{d}$) term

$$R_{d}(f_{x})=\frac{M_{\mathrm{AC}}(f_{x})}{M_{\mathrm{AC},\text{phantom}}(f_{x})}R_{d,\text{phantom}}(f_{x}).$$ (2)

The next step of processing included an inverse lookup table (LUT) scheme to match the measured pairs of $R_{d}$ to a pair of $\mu_{a}$ and ${\mu_{s}}^{\prime }$ values. Here, the LUT for $R_{d}(f_{x})$ at $f_{x}=0$ and $0.3\;{\mathrm{mm}}^{-1}$ was generated using the same WMC code mentioned above with $\mu_{a}$ range of 0.0001 to $0.2\;{\mathrm{mm}}^{-1}$ and ${\mu_{s}}^{\prime }$ range of 0.001 to $3\;{\mathrm{mm}}^{-1}$ for 512 linearly spaced values. We employed a rapid LUT method using an interpolation scheme for scattered data from a built-in MATLAB function (scatteredInterpolant) to generate an inverse LUT for a pair of $\mu_{a}$ and ${\mu_{s}}^{\prime }$ at each pixel.

After the extraction of $\mu_{a}$ and ${\mu_{s}}^{\prime }$, we calculated chromophore concentrations using $\mu_{a}$ at all three wavelengths. Here, we calculated an inverse matrix of extinction coefficients at each wavelength for ${\mathrm{HbO}}_{2}$ and HbR, which was then multiplied with $\mu_{a}$ at each pixel. In Matlab, this method is analogous to least square fitting when the matrices have mismatched dimensions. Finally, we used HbR and ${\mathrm{HbO}}_{2}$ concentration to calculate physiologically relevant parameters, such as HbT and ${\mathrm{StO}}_{2}$ using their definitions

$$\mathrm{HbT}={\mathrm{HbO}}_{2}+\mathrm{HbR},$$ (3)

$${\mathrm{StO}}_{2}=\frac{{\mathrm{HbO}}_{2}}{\mathrm{HbT}}\times 100.$$ (4)

### 4.2 LSI Processing Pipeline

We employed a typical spatial speckle contrast algorithm with a 5x5 sliding window for analysis.^9^ We used MATLAB to perform the computations using the following equation:

$$K=\frac{\sigma (I)}{\left\langle I\right\rangle },$$ (5)

were σ(I) and ⟨I⟩ are the standard deviation and mean values of the intensity, respectively, within the sliding window. Then, the center pixel of the window was assigned the contrast value K. We further converted speckle contrast K to speckle flow index (SFI) using the following simplified speckle imaging equation:^31^^,^^32^

$$\mathrm{SFI}=\frac{1}{2TK^{2}},$$ (6)

where T is the exposure time of the camera. Our previous publications have shown that SFI closely approximates $\frac{1}{\tau_{c}}$, where $\tau_{c}$ is the decorrelation time of the electric field autocorrelation function.^31^^,^^32^ In most laser speckle neuroimaging literature, $\frac{1}{\tau_{c}}$ is considered an approximation of CBF.^5^^,^^6^^,^^9^

For each LSI measurement, β is a normalization factor that takes into account the mismatch between detector and speckle sizes and polarization. Typically, β can be estimated using speckle contrast K (where $\tau_{c}\gg T$) measured from a static phantom as $\beta \approx K_{\text{phantom}}^{2}$.^9^ We then use β to correct for SFI

$${\mathrm{SFI}}_{\beta -\text{corrected}}=\beta \cdot \mathrm{SFI}.$$ (7)

### 4.3 Obtaining Absolute Flow Values by Correcting for Optical Properties

In this section, we describe in detail a protocol used to obtain an absolute measurement of flow by correcting speckle contrast K with optical properties. We first started with an equation relating K to electric field autocorrelation $G_{1}$ as described by Mazhar et al.^15^

$$K^{2}=\frac{\frac{2\beta }{T}\int_{0}^{T}G_{1}^{2}(\tau )(1-\frac{\tau }{T})d\tau }{G_{1}^{2}(\tau =0)}.$$ (8)

The associated solution for $G_{1}$ in the spatial frequency domain is

$$G_{1}(\tau )=\frac{3P_{0}A\frac{\mu_{s}^{\prime }}{\mu_{tr}}}{\left(\frac{\mu_{\mathrm{eff}}^{\prime }}{\mu_{tr}}+1)(\frac{\mu_{\mathrm{eff}}^{\prime }}{\mu_{tr}}+3A\right)}.$$ (9)

In Eq. (9), $\mu_{tr}=\mu_{a}+{\mu_{s}}^{\prime }$ is the transport coefficient, and $P_{0}$ is the illumination power. Here, A was defined as

$$A=\frac{1-R_{\mathrm{eff}}}{2(1+R_{\mathrm{eff}})};\;\text{where}\;R_{\mathrm{eff}}\approx 0.0636n+0.668+\frac{0.710}{n}-\frac{1.440}{n^{2}}.$$ (10)

In the spatial frequency domain, ${\mu_{\mathrm{eff}}}^{\prime }$ is defined as

$$\mu_{\mathrm{eff}}^{\prime }=\;\sqrt{\mu_{\mathrm{eff}}^{2}+(2\pi f_{x}{)}^{2}};\;\text{where}\;\mu_{\mathrm{eff}}=\sqrt{3(\mu_{a}+\frac{1}{3}\mu_{s}^{\prime }{\left(\frac{2\pi n}{\lambda }\right)}^{2}(6D_{b}\tau ))\mu_{tr}}.$$ (11)

In this equation, $f_{x}$ is the spatial frequency of the illumination pattern; n is the refractive index; λ is the LSI wavelength; and $D_{b}$ is the “effective” Brownian diffusion coefficient describing the motion of scatterers.^33^ This equation is often used in dynamic light scattering theory to describe the mean-squared displacement of moving particles. Furthermore, the Brownian model yielded a better fit for the observed correlation decay curves in various tissue types.^34^ In this work, we assumed that LSI illumination is planar; thus, $f_{x}$ is assumed to be 0.

**Fig. 2:**
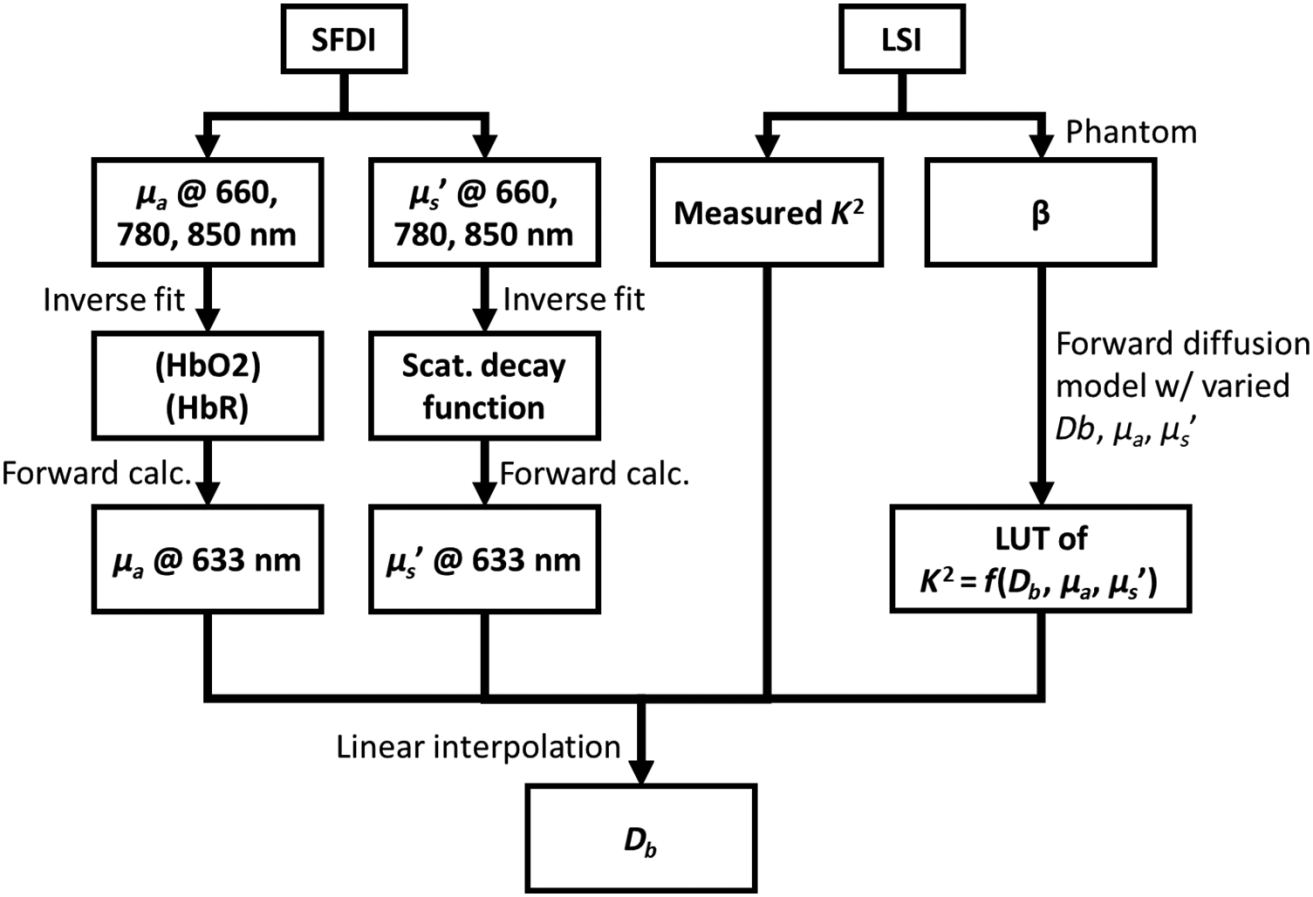
Flow chart of the method of obtaining absolute flow value using the Brownian diffusion coefficient $D_{b}$ from measured K, $\mu_{a}$, and ${\mu_{s}}^{\prime }$. Here, a β factor was approximated using a static silicone phantom.

Our goal was to retrieve $D_{b}$ using $\mu_{a}$, ${\mu_{s}}^{\prime }$, and speckle contrast K. The overall schematic of deriving $D_{b}$ from measured parameters is described in Fig. 2. First, we used concentrations of ${\mathrm{HbO}}_{2}$ and HbR determined with SFDI to calculate $\mu_{a}$ at 633 nm. Then, to determine ${\mu_{s}}^{\prime }$ at 633 nm, we fit an exponential function in the form of the Mie-scattering exponential decay function^35^

$$\mu_{s}^{\prime }(\lambda )=A{\left(\frac{\lambda }{850}\right)}^{b}↔\log(\mu_{s}^{\prime }(\lambda ))=\log(A)+b\cdot \log(\frac{\lambda }{850}).$$ (12)

To perform the fit more efficiently, we rewrote the decay function, as shown in Eq. (12). We then solved for log(A) and b using MATLAB “\” operator using the ${\mu_{s}}^{\prime }$ measured at 660, 780, and 850 nm. From log(A) and b, we obtained a value of ${\mu_{s}}^{\prime }$ at 633 nm with a forward solution. We used a β factor to create a LUT of $K^{2}$ values as a function of $D_{b}$, $\mu_{a}$, and ${\mu_{s}}^{\prime }$ by numerically integrating Eq. (8). The values of $\mu_{a}$ and $\mu_{s}n$ in the LUT were chosen to cover the range that included the values at 633 nm (i.e., $\mu_{a}$ from 0.0001 to $0.3\;{\mathrm{mm}}^{-1}$ and ${\mu_{s}}^{\prime }$ from 0.001 to $3\;{\mathrm{mm}}^{-1}$ with 128 equally spaced values), and the range of $D_{b}$ was set to 0 to $20^{-6}\;{\mathrm{mm}}^{2}/s$ with 256 equally spaced values. Using the MATLAB function scatteredInterpolant, we then obtained the value of $D_{b}$ from the set of measured K, $\mu_{a}$, and ${\mu_{s}}^{\prime }$ at 633 nm.

## 5 Characterization

For each of the experiments listed below, the system was turned on and warmed up for at least 10 min before imaging. This allowed for the LEDs in the SFDI system to reach a steady-state temperature, which was expected to minimize longitudinal drift in the illumination.

### 5.1 Flow Sensitivity Using LSI with a Flow Phantom Setup

**Fig. 3:**
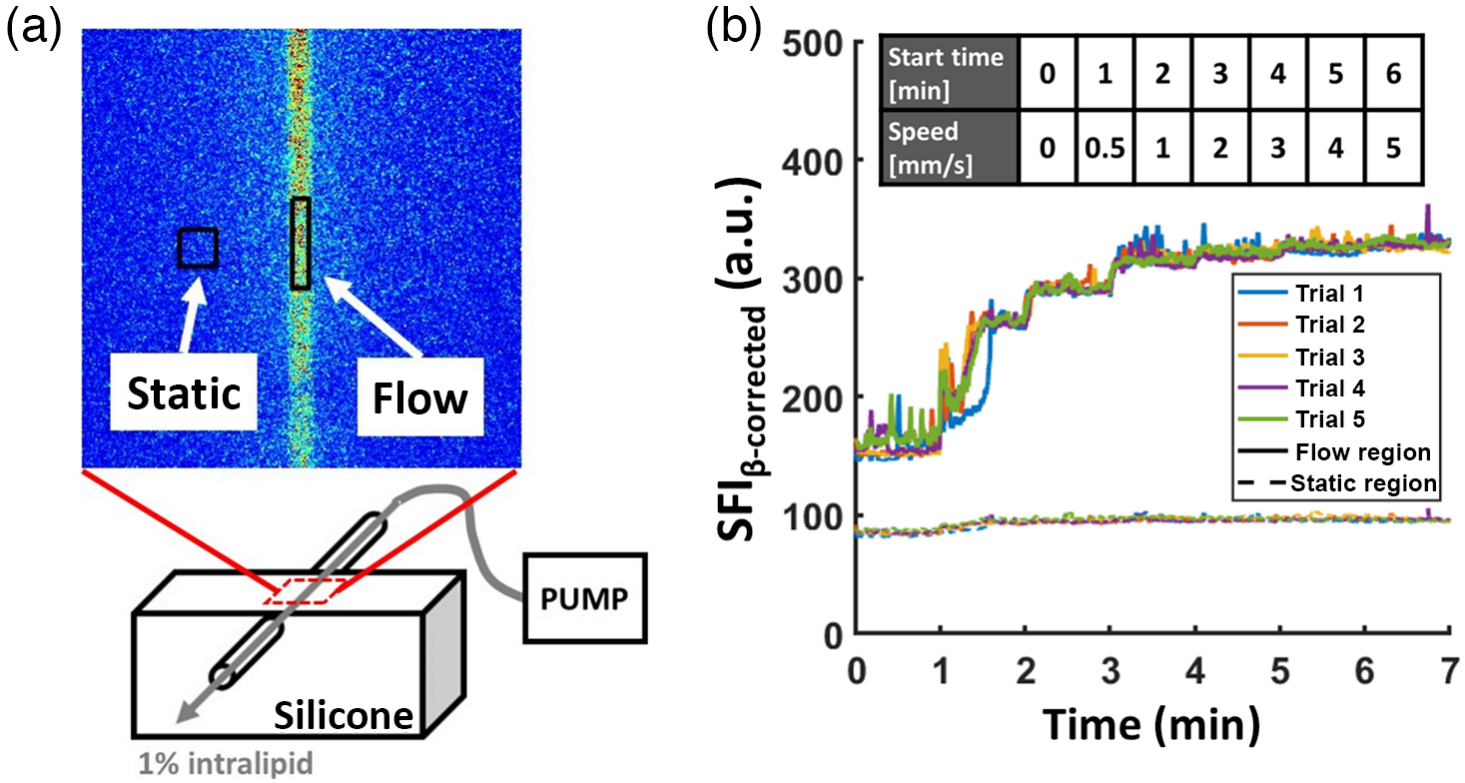
Flow phantom experiment to characterize the sensitivity of the LSI arm. (a) A schematic of the silicone flow phantom with an embedded glass capillary pipet tube where a solution of 1% intralipid was flowed at different speeds using a syringe pump. A representative flow map of the captured FOV was also shown with the chosen ROI (black rectangles) for static and flow regions. (b) Time-course data of the experiment where the flow speed was increased at each 1-min interval (top table). The time course of ${\mathrm{SFI}}_{\beta \text{-corrected}}$ within the flow region showed an increasing pattern with increasing speed between 0 and ∼4  mm/s. A plateau pattern was observed at higher speeds (>4  mm/s). For the static region, ${\mathrm{SFI}}_{\beta \text{-corrected}}$ showed no change during the experiment.

We used a flow phantom setup to characterize the flow sensitivity of the LSI arm of QHI as previously described.^36^^,^^37^ The static part of the phantom was made using silicone with no added absorbers and ${\mathrm{TiO}}_{2}$ as scatterers. The optical properties of the static portion were measured using SFDI, with $\mu_{a}=0.0049$, 0.0059, and $0.0062\;{\mathrm{mm}}^{-1}$ and ${\mu_{s}}^{\prime }=0.87$, 0.67, and $0.60\;{\mathrm{mm}}^{-1}$ at 660, 780, and 850 nm, respectively. A 25  μL glass capillary pipet tube (Kimble 71900, inner radius ∼330  μm) was embedded in the silicone phantom so that the tube was flush with the surface of the phantom [Fig. 3(a)]. A 1% intralipid solution was prepared and infused into the capillary tube using a precision pump (70-4504, Harvard Apparatus, Massachusetts, United States) at incremental speeds of 0, 0.5, 1, 2, 3, 4, and 5  mm/s for 60 s at each speed. The imaging session lasted 7 min in total. We repeated the experiment for 5 trials, with 15 min of wait time between each trial. This wait time is implemented to ensure that the flowing fluid is restored to a baseline state.

Two regions of interest (ROIs) were chosen over the dynamic and static region of the phantoms and median values of ${\mathrm{SFI}}_{\beta \text{-}\text{corrected}}$ were calculated at each imaging time point and filtered using a 50-frame (∼1  s) moving average filter. The resulting time course data sets are shown below in Fig. 3(b). Here, the dynamic ROI showed the expected increase in ${\mathrm{SFI}}_{\beta \text{-}\text{corrected}}$ values with increasing speed, with the sensitivity to flow reaching a plateau starting at ∼4  mm/s, similar to what has been reported previously with T=10  ms.^31^ The static ROI showed no change in SFI despite the increasing speeds.

### 5.2 Optical Property Accuracy Assessment of SFDI

**Fig. 4:**
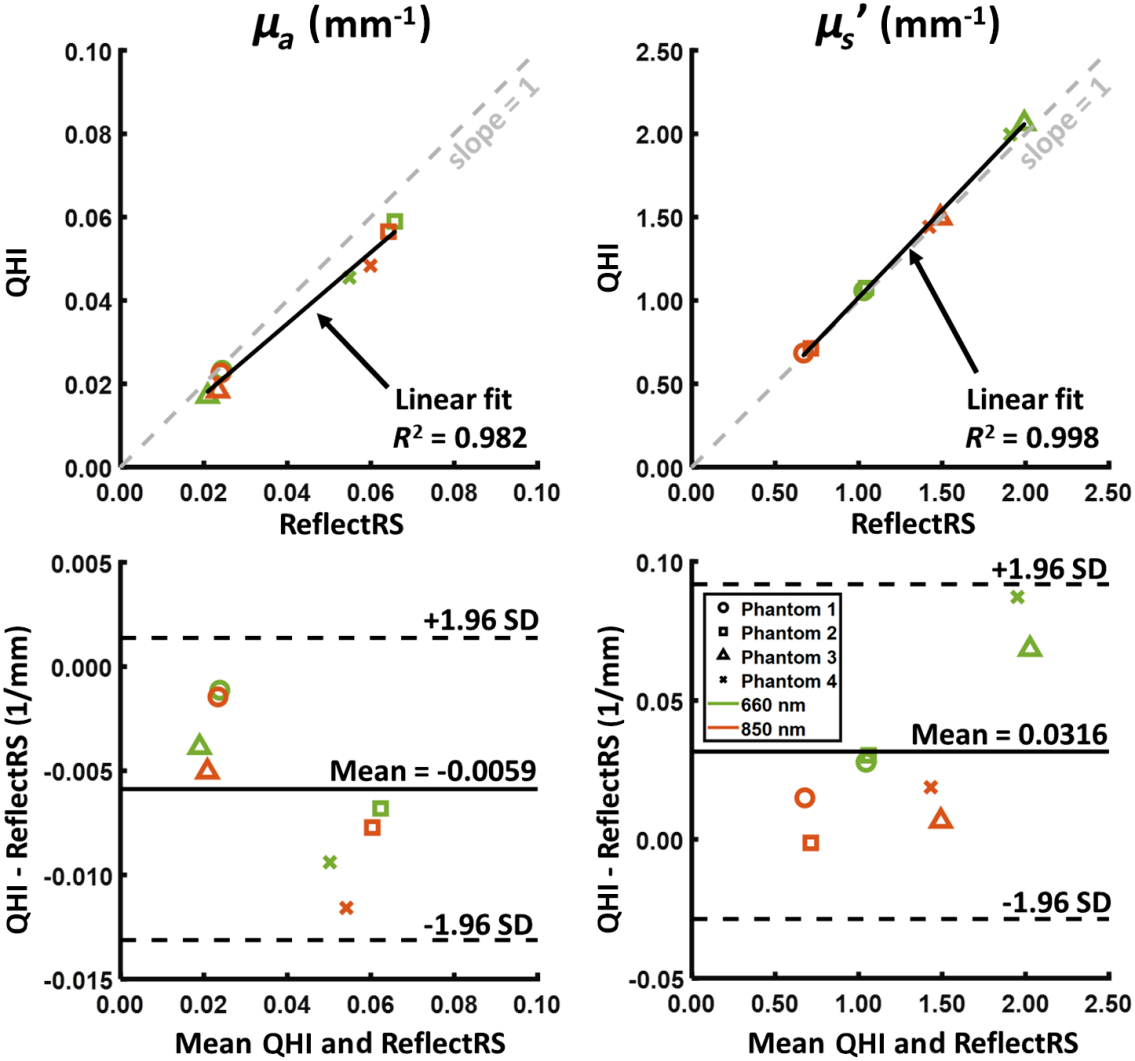
Correlation (top) and Bland-Altman plots (bottom) between QHI and a commercial ReflectRS system from Modulim of four static silicone phantoms. Here, we compared the median values obtained from the chosen ROI. Only 660 nm and 850 nm wavelengths were compared, as 780 nm is not available with the ReflectRS. For the correlation plot, $R^{2}$ values were also listed for the linear fit.

We fabricated four static silicone phantoms and compared optical property measurements between the SFDI arm of QHI with a commercial SFDI system. The phantoms were made using a base of silicone, ${\mathrm{TiO}}_{2}$ powder as scatterers, and India ink as absorbers. One phantom had a specific set of $\mu_{a}$ and ${\mu_{s}}^{\prime }$, and the other three phantoms were fabricated to have the following optical property values relative to the first phantom: (1) same $\mu_{a}$, $2\times {\mu_{s}}^{\prime }$; (2) same ${\mu_{s}}^{\prime }$, $2\times \mu_{a}$; and (3) $2\times \mu_{a}$ and $2\times {\mu_{s}}^{\prime }$. Here, the values of $\mu_{a}$ and ${\mu_{s}}^{\prime }$ measured using QHI were compared with measurements from a commercial SFDI system (ReflectRS, Modulim, California, United States). A common circular ROI with a diameter of 3.3 mm was selected and median values of the optical properties were extracted. We performed a linear regression analysis using the median $\mu_{a}$ and ${\mu_{s}}^{\prime }$ values from the four phantoms measured using QHI and the ReflectRS for 660 and 850 nm. This wavelength choice resulted from the fact that the ReflectRS does not have a 780 nm LED. Here, we observed a high linear correlation between QHI and the ReflectRS ($R^{2}>0.98$) for both $\mu_{a}$ and ${\mu_{s}}^{\prime }$ (Fig. 4). Using a Bland–Altman plot, we observed a mean difference of −0.0059 and $0.0316\;{\mathrm{mm}}^{-1}$ for $\mu_{a}$ and ${\mu_{s}}^{\prime }$, respectively (Fig. 4). Collectively, these data suggest that QHI slightly underestimated $\mu_{a}$ and slightly overestimated ${\mu_{s}}^{\prime }$; however, this discrepancy was linear for various combinations of $\mu_{a}$ and ${\mu_{s}}^{\prime }$ (as shown through high $R^{2}$). One reason for this discrepancy could be the mismatched FOV of the two systems, as QHI had a much smaller total FOV (i.e., 1×1  cm versus 20×15  cm, respectively, ∼300× smaller) due to its higher magnification, with an increased sensitivity to any heterogeneities within the phantom (e.g., locally dense regions of ${\mathrm{TiO}}_{2}$), which could skew the median value.

### 5.3 Intra-Day Inter-Session Precision Measurements of Static Silicone Phantom

**Fig. 5:**
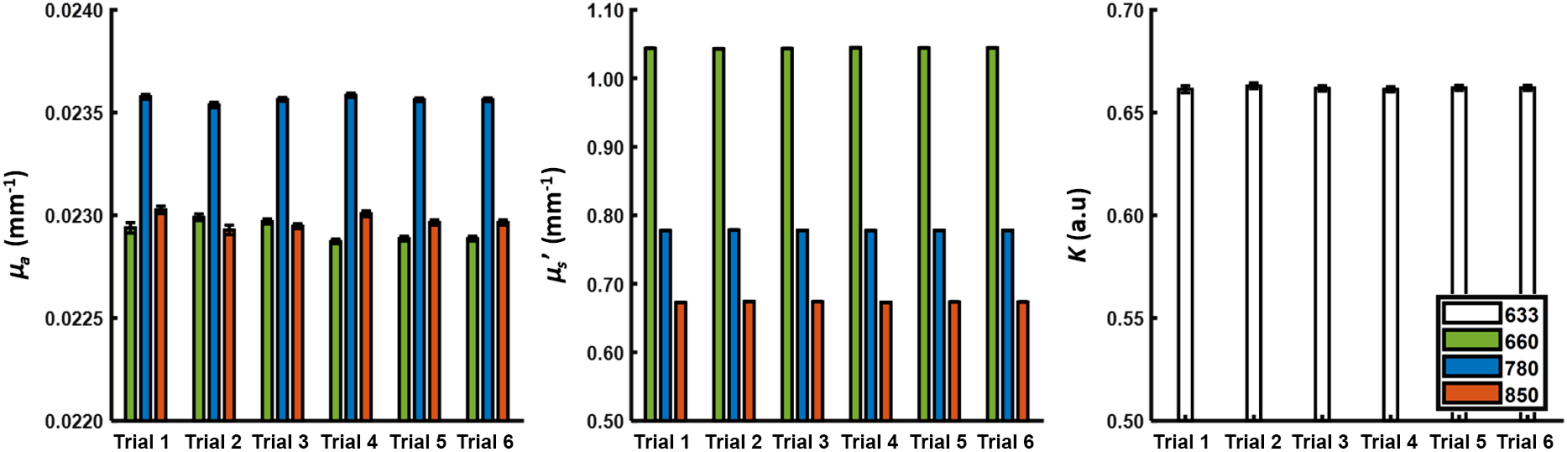
Measurements of QHI precision were performed for six trials (5 min each) within the same day on the same static silicone phantom (i.e., phantom 1). The mean and standard deviation are plotted here. The recorded values for $\mu_{a}$, ${\mu_{s}}^{\prime }$, and K and the calculated coefficient of variation across all trials were shown in Table S1 in the Supplementary Material.

To investigate the stability of the system during a session and among multiple sessions within one day of imaging, we imaged phantom 1 in six separate trials. Here, each trial lasted 5 min, and the time between the start of each trial was 30 min. The total length of the experiment was 3 h. A circular diameter with a radius of 200 pixels (∼1.6  mm) was chosen at the center of the FOV and median values of $\mu_{a}$, ${\mu_{s}}^{\prime }$, and K were recorded at each respective wavelength at each time point. A whisker bar graph of all median values for the five minutes of imaging for each measured optical parameter at each respective wavelength is shown in Fig. 5. We also calculated the coefficient of variation across all trials using the mean value of each trial’s time course. For SFDI measurements of $\mu_{a}$ and ${\mu_{s}}^{\prime }$ of all wavelengths, the standard deviations for each of the trials were <0.001 while the coefficient of variation across all trials was <0.5%. For LSI measurements of K, we observed standard deviations ranging from 0.0014 to 0.0013 for each of the trials with a coefficient of variation across all trials of 0.16%. These values were listed in Table S1 in the Supplementary Material and indicated good intraday stability of the system.

### 5.4 Longitudinal Precision Measurement of Static Silicone Phantom

**Fig. 6:**
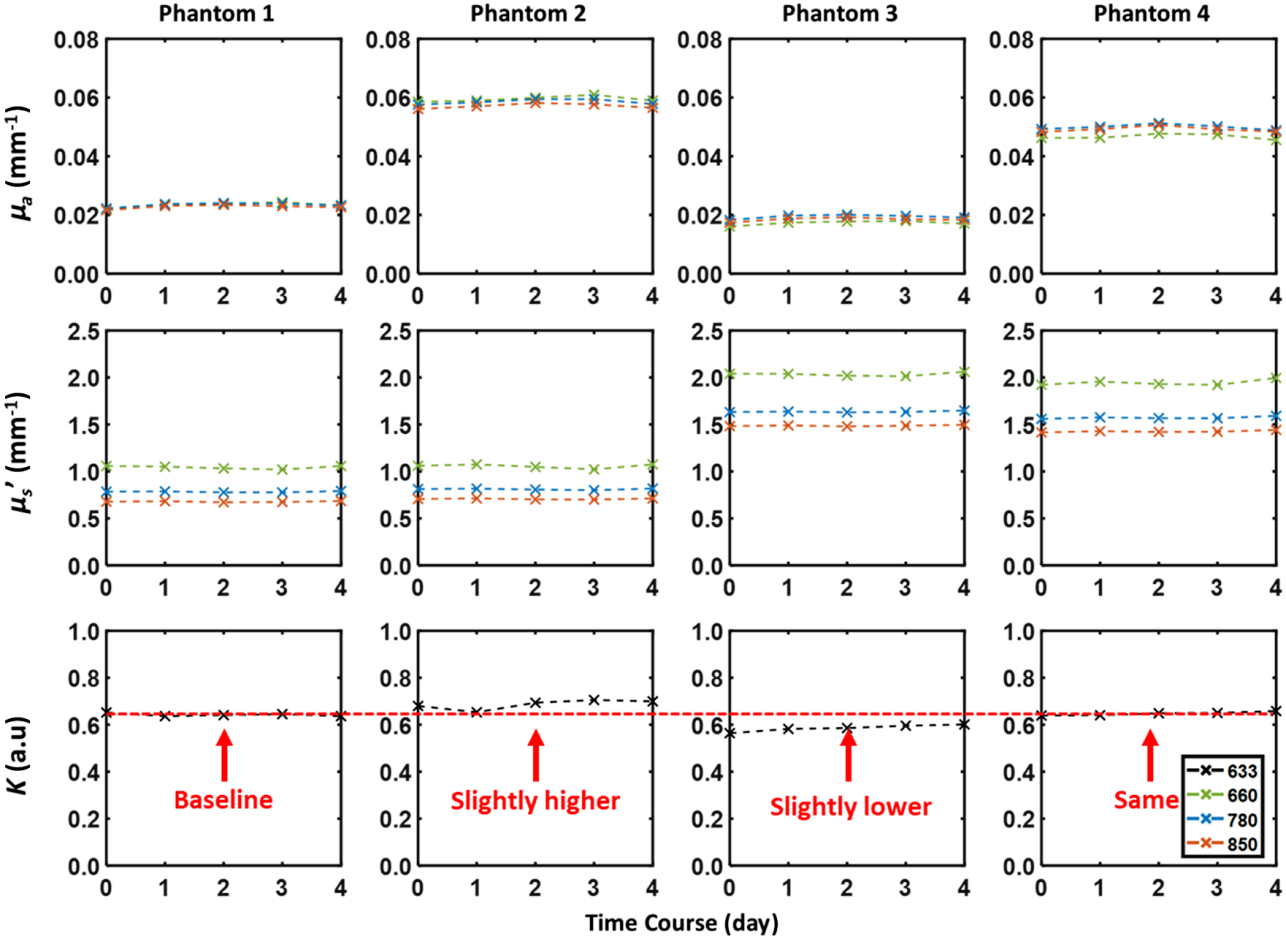
Measurements of QHI precision over five days. The median values within the selected ROI for each day are plotted and further used in the analysis. The recorded values for $\mu_{a}$, ${\mu_{s}}^{\prime }$, and K and the calculated coefficient of variation across all five days are shown in Tables S2–S4 in the Supplementary Material. K is higher for the phantom with higher $\mu_{a}$ (phantom 1 versus phantom 2) and lower for the phantom with higher ${\mu_{s}}^{\prime }$ (phantom 1 versus phantom 3). With higher $\mu_{a}$ and ${\mu_{s}}^{\prime }$ (phantom 4), K is similar to that of phantom 1. This agrees with the findings of Mazhar et al.^15^

Using the four static silicone phantoms listed above, we conducted longitudinal precision measurements over 5 days. A single full set of LSI and SFDI frames (a total of 18 frames) was taken and analyzed. A circular ROI with a radius of 200 pixels (∼1.6  mm) was chosen at the center of the FOV and median values of $\mu_{a}$, ${\mu_{s}}^{\prime }$, and K were determined at each respective wavelength. These median values across the 5 days were then used to calculate a coefficient of variation for each measured parameter. Here, we noted that all coefficients of variations were <4% (Fig. 6, Tables S2–S4 in the Supplementary Material), indicating good precision of the system across multiple days. Here, K will theoretically be 1 for a completely static phantom. However, the measured K from the four phantoms ranged between 0.5 and 0.7 due to potential environmental, experimental, or instrumental sources. For phantom 2, K increased with increasing $\mu_{a}$ values compared to those of phantom 1 due to the decreasing probability of detection of a photon hitting a dynamic scattering event (i.e., a dynamic photon). On the contrary, phantom 3 showed a decrease in contrast K with increasing ${\mu_{s}}^{\prime }$ due to the increasing probability of detection of a dynamic photon. Finally, phantom 4 showed similar K values to those of phantom 1 when both increasing $\mu_{a}$ and ${\mu_{s}}^{\prime }$. The measured K values differed across the four phantoms due to $\mu_{a}$ and ${\mu_{s}}^{\prime }$ changes as expected from Eqs. (8)–(11) (Fig. 6, red dotted line for K graphs).

## 6 *In-vivo* Neuroimaging Demonstration

We then assessed the *in vivo* efficacy of the system in two experiments: (1) measuring intra-animal changes in hemodynamics during hypercapnia and (2) inter-animal comparison of resting-state CBF measured using either SFI or $D_{b}$. For both experiments, the correction of CBF using the derived optical properties showed impactful changes.

### 6.1 Intra-Animal CBF Measurement During Hypercapnia Gas Challenge

**Fig. 7:**
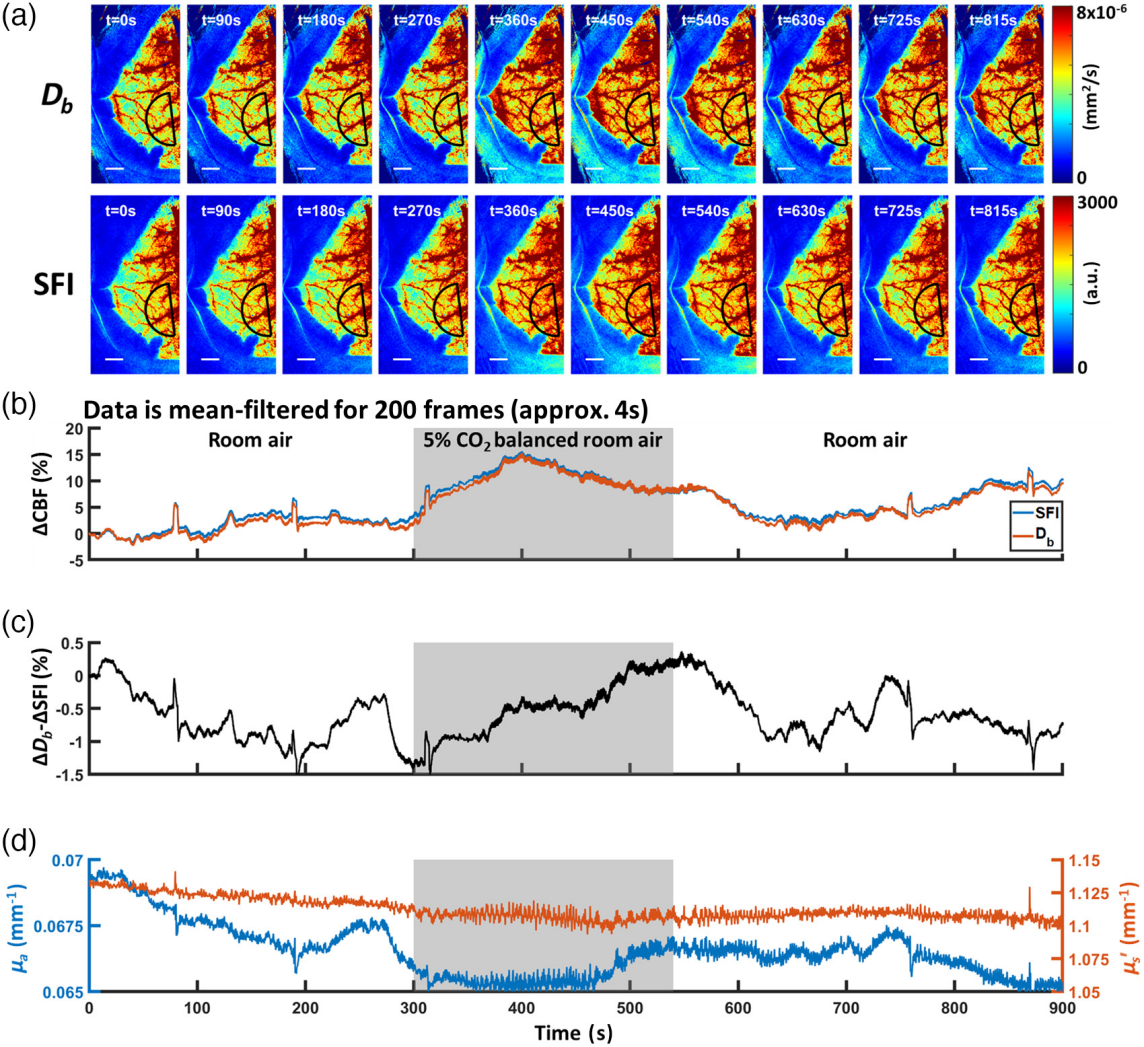
CBF dynamics slightly differentiate between $D_{b}$ and SFI measurements for a mouse undergoing a hypercapnia gas challenge. (a) Representative images of $D_{b}$ and SFI during hypercapnia with the chosen semicircular ROI (black) on the left hemisphere. Scale bars are 1 mm. b) CBF measurements normalized to t=0 where both SFI and $D_{b}$ analysis was performed. Median data are chosen from the left hemisphere to extract the time course of SFI and $D_{b}$ values. The data is further filtered using a moving filter of 200 frames (∼4  s). (c) A difference of −1.5 to 0.5% between the normalized $D_{b}$ and SFI curves is observed throughout the gas challenge experiment. (d) Optical properties derived at 633 nm showing a slight decrease in both $\mu_{a}$ and ${\mu_{s}}^{\prime }$ across the time course.

To assess the impact of the processing methods on dynamic measurements, we subjected a female mouse (C57BL/6, 5 months old) to a cranial window preparation for hypercapnia. Anesthesia was initiated at 4% isoflurane using an induction chamber and the animal was maintained at 2% for the surgical period at 1  L/min flow rate. For the gas challenge, we used 1.5% isoflurane to maintain anesthesia and the following experimental protocol: 5 min of baseline with balance room air, 4 min of 5% ${\mathrm{CO}}_{2}$ and balance room air, followed by 6 min of the initial isoflurane-room air condition. We extracted the time course of SFDI and LSI data using a semicircular ROI on the left hemisphere (radius of 175 pixels, ∼1.4  mm). The ROI was chosen with its position along the midpoint between bregma and lambda and an offset of 100 pixels (∼0.8  mm) from the midline. The representative CBF maps of $D_{b}$ and SFI with the chosen ROI can be seen in Fig. 7(a). We then normalized $D_{b}$ and SFI measurements to time t=0 and obtained a percentage ΔCBF. Due to normalization, β correction is not required for SFI measurements. The time courses were then filtered using a moving average of 200 data points, approximating a 4-s period. In this experiment, as expected we observed a hyperemic response during the hypercapnic period [Fig. 7(b)]. However, there was a slight difference between the filtered SFI and $D_{b}$ time courses [Fig. 7(c)]. This difference ranged from −1.5 to 0.5% during the experimental period, corresponding to the range of changes of $∼0.005\;{\mathrm{mm}}^{-1}$ (∼7%) in $\mu_{a}$ and $∼0.025\;{\mathrm{mm}}^{-1}$ (∼2%) in ${\mu_{s}}^{\prime }$ [Figs. 7(c) and 7(d)]. This decrease in $\mu_{a}$ and ${\mu_{s}}^{\prime }$ resulted in a minimal change in CBF dynamics between $D_{b}$ and SFI measurements. In fact, Eq. (9) predicts that $K^{2}$ remains constant with a defined $D_{b}$ if the changes of $\mu_{a}$ and ${\mu_{s}}^{\prime }$ happen with the same multiplicative factor. For example, if $\mu_{a}$ and ${\mu_{s}}^{\prime }$ both increase by 50%, $K^{2}$ stays the same. Intuitively, the loss of correlation due to scattering cancels the increase of correlation from the loss of the traveling photon due to absorption. However, in the case where only one of the two optical properties remains constant while the other is changing, $K^{2}$ will change accordingly. A simulation result of this phenomenon was shown in Fig. S1 in the Supplementary Material.

### 6.2 Inter-Animal CBF Measurement at Resting State

We measured the resting-state CBF of a cohort of mice (n=13, B6SJL background, 15 months old). Anesthesia was initiated using 4% isoflurane (balance oxygen) within an enclosed chamber and the animals were maintained at 2% isoflurane (balance room air) at 1  L/min flow rate. We then performed an acute intact-skull cranial window surgical procedure to gain access to the cortical layer of the brain.^38^ The scalp was shaved, depilated, and sterilized with alcohol and povidone-iodine wipes. We then performed a midline incision, retracted the scalp, and removed the fascial layer to expose the relatively transparent skull. The window was then filled with saline and a coverslip was applied to create a translucent skull for imaging the cortical surface.

**Fig. 8:**
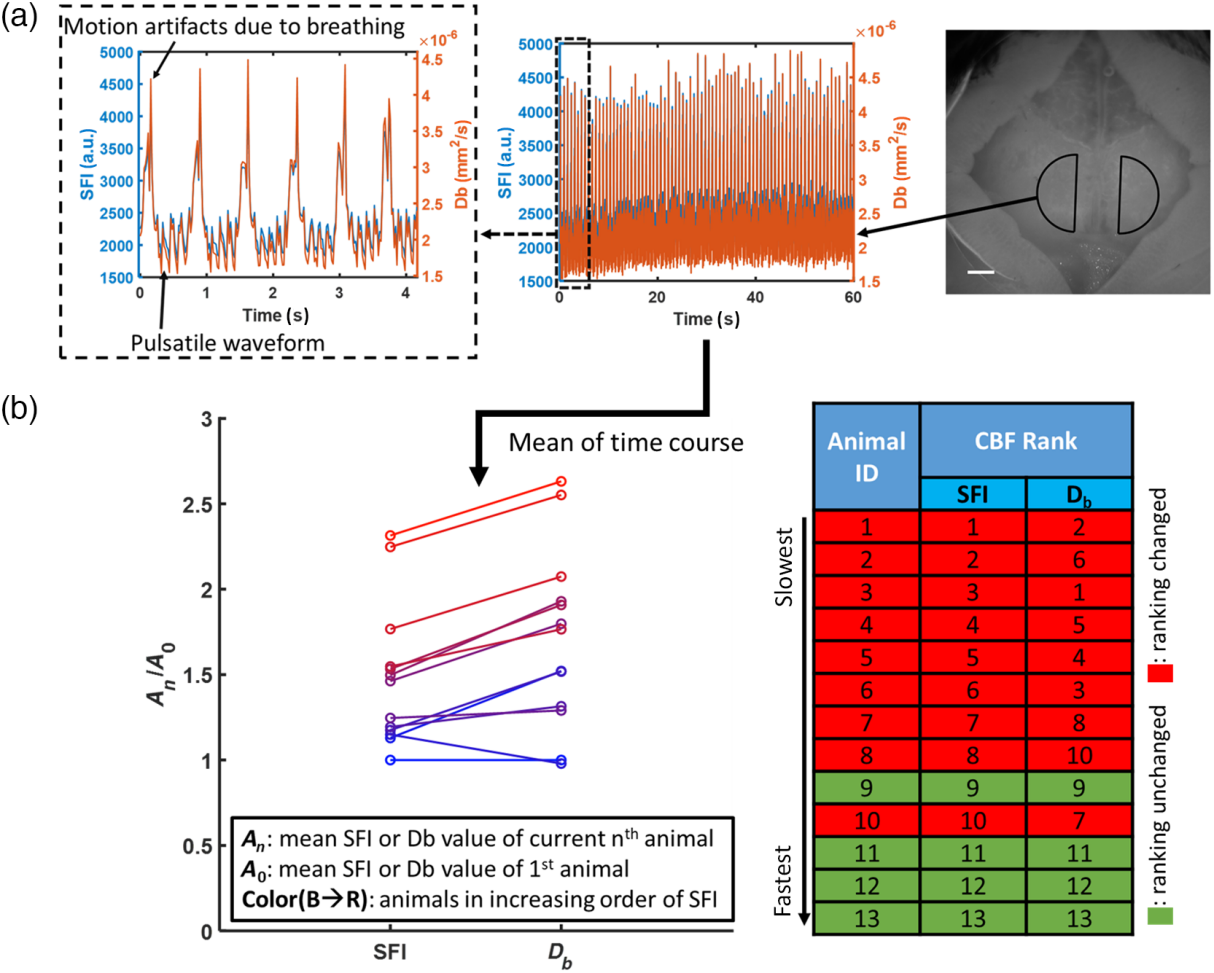
Baseline CBF measurements from 13 mice (2% isoflurane for 1 min) highlighted the effect of optical properties on the interpretation of CBF measurements using LSI. (a) Median values of data obtained from the left hemisphere were used to generate time course data for the 1-min imaging period. Both the pulsatile CBF waveform and breathing-related motion artifacts could be resolved. On the representative image on the right, the scale bar is 1 mm. (b) Mean values of the SFI time course during each imaging period were then used to rank the animals in ascending order (blue to red circles). Animal IDs (1 to 13) were then assigned based on this ranking. Here, the $D_{b}$ values differed from SFI in ranking for nine out of 13 animals.

Resting-state data were taken for one minute at 2% isoflurane and a common semi-circular ROI (radius of 175 pixels, ∼1.4  mm) on the left hemisphere was chosen [Fig. 8(a)]. The ROI was chosen with its position along the midpoint between bregma and lambda and an offset of 100 pixels (∼0.8  mm) from the midline. To calculate SFI and $D_{b}$, we chose the median value within the ROI to generate time course data for all measured parameters (e.g., K, $\mu_{a}$, and ${\mu_{s}}^{\prime }$). We applied the processing methods discussed above to generate optical properties at 633 nm and convert SFI to $D_{b}$ [Fig. 8(a)]. Here, we only applied the processing methods on the 1-min time-course data and not on every pixel within the FOV.

To assess the impact of optical property correction on CBF, we calculated the mean of the time courses of SFI and $D_{b}$. An initial ranking of resting-state CBF was generated using SFI values [Fig. 8(b), blue to red for lowest to highest SFI measurements]. The animal IDs were also assigned using this ordering (i.e., animal 1 was the one with the lowest SFI). To facilitate a more direct comparison of SFI and $D_{b}$, we normalized the individual SFI and $D_{b}$ values for all animals to those of animal 1. Due to normalization, β correction is not required for SFI measurements. Using this comparison method, we observed that the ranking of CBF using $D_{b}$ differs from that using SFI. Specifically, 9 out of 13 animals showed a change in the ranking [Fig. 8(b)], thus highlighting the potential effect of optical properties on speckle-based measurements of CBF.

**Fig. 9:**
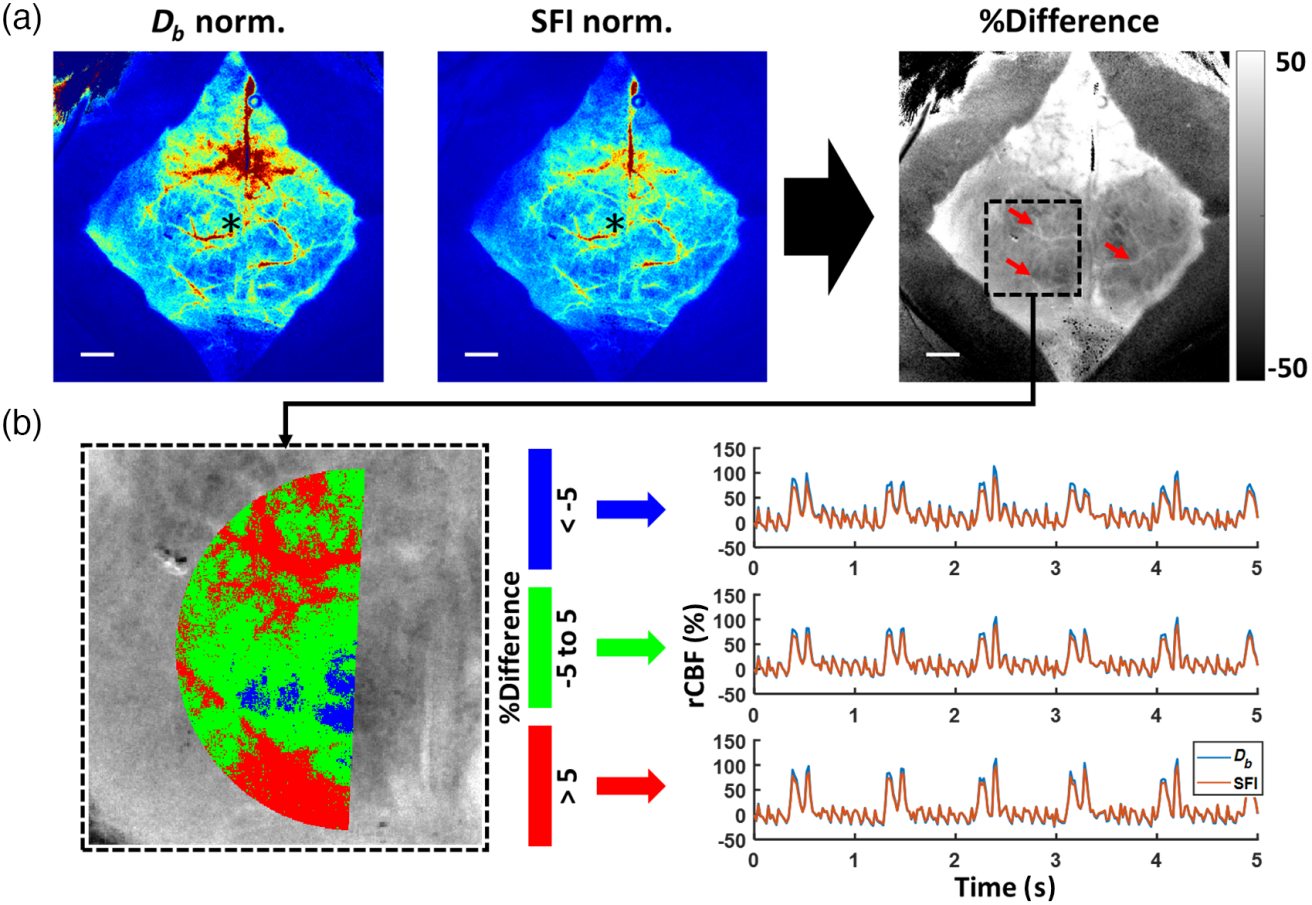
On one representative animal, $D_{b}$, when compared to SFI map, showed a spatial difference in CBF measurements but not in temporal dynamics. (a) Representative normalized maps of $D_{b}$ and SFI highlight the spatial difference in estimated CBF. Individual pixels in each image were normalized relative to the respective center pixel (*). A percentage difference map was generated using $\%\text{Difference}=(D_{b,\text{norm}}-{\mathrm{SFI}}_{\text{norm}})/{\mathrm{SFI}}_{\text{norm}}\times 100$. $D_{b}$ showed increased blood flow in large vessels (red arrows) compared with SFI. Scale bars are 1 mm. (b) Within the chosen semicircular ROI, temporal dynamics (i.e., rCBF) did not show notable differences between SFI and $D_{b}$ measurements among regions with decreasing (<−5%), minimally changing (−5 to 5%), and increasing (>5%) spatial differences. The power spectrum of both $D_{b}$ and SFI (not shown here) showed negligible differences in the pulsatile waveforms.

Figure 9(a) showed representative normalized CBF images that were generated using a pixel-wise correction algorithm. All pixels in the $D_{b}$ and SFI images were normalized to the respective values of the center pixel in each image. We also calculated a percentage difference between normalized $D_{b}$ and SFI maps [Fig. 9(a)] and noted higher CBF values in larger vessels (red arrows) in the $D_{b}$ image. Currently, this pixel-by-pixel method of correction is currently not applied to time-course analysis due to the long processing time for images with such high pixel resolution (1.44 MP). We further investigated if these spatial differences would also yield a change in temporal dynamics. On the same representative animal, we used the percentage difference map to define three groups of pixels within the chosen ROIs mentioned in Fig. 8. These three groups correspond to the pixels that showed a decrease [<−5%, Fig. 9(b) blue], minimal change [−5 to 5%, Fig. 9(b) green], or increase [>5%, Fig. 9(b) red] in the spatial percentage difference map. We then calculated rCBF as relative CBF from t=0 for $D_{b}$ and SFI time courses using the median value of all the pixels within each respective region. As shown in Fig. 9(b) below, the $D_{b}$ and SFI time courses showed similar dynamics at the resting state for all regions. The power spectra, not shown here, did not yield notable differences in the pulsatile signal.

## 7 Discussion

This work outlined the design of a QHI system that combined LSI and SFDI to achieve quantitative CBF measurements independent of optical properties. $D_{b}$ measurements of one mouse under hypercapnia showed a slight difference from SFI one. Additionally, the *in vivo* resting-state measurements showed a change in the ranking of CBF under anesthesia after correcting LSI measurements using optical properties obtained from SFDI. This discrepancy may impact statistical analyses of proposed hypotheses in experiments that involve comparing the CBF of several groups (e.g., disease-state versus wild-type). The results further illustrate the need to correct for the effects of optical properties on speckle contrast measurements.

In LSI literature, relative CBF (i.e., normalized to baseline or t=0) is the most commonly used quantitation method.^5^^,^^9^^,^^13^ Attempts to enable further quantitative measurements have focused on utilizing the dynamics at various exposure times, also known as multi-exposure speckle imaging (MESI), to account for the variability of the optical setup (i.e., β) and the presence of static scatterers.^39^^–^^41^ However, the theory of MESI only focused on fitting the shape of the contrast-versus-exposure-time functions and did not account directly for optical properties. Meanwhile, our method utilized the solution of the correlation diffusion equation ^42^ to relate speckle contrast K to $\mu_{a}$, ${\mu_{s}}^{\prime }$, and $D_{b}$. This method allows for directly estimating $D_{b}$ using the measured LSI and SFDI data. In addition to neuroimaging, QHI can be applied to other applications, such as burn assessment and peripheral blood flow monitoring.^17^^,^^43^^,^^44^ In these applications, QHI enables quantitative blood flow measurements independent from potential confounding factors such as pigmentation (i.e., variation in absorption) and collagen denaturation (i.e., changes in scattering) in burn wounds. Despite its advantages, the system is not free from limitations, as discussed below.

### 7.1 Current Hardware Limitations

The system has a 50 Hz raw frame rate at 1200×1200  pixel resolution of a co-aligned 10  mm×10  mm FOV. This frame rate enabled an effective 50 Hz and 2.8 Hz frame rate for LSI and SFDI arms, respectively. Using *in vitro* phantoms, for sensitivity and accuracy measurements, we showed that QHI can detect differences in LSI flow speed from 0 to 4  mm/s while also achieving a strong linear correlation of optical properties (i.e., $\mu_{a}$ and ${\mu_{s}}^{\prime }$) measurements with a commercial SFDI system ($R^{2}>0.95$). We observed a slight underestimation of $\mu_{a}$ and a slight overestimation of ${\mu_{s}}^{\prime }$ between QHI and the commercial system.

The openSFDI guide suggested that the exposure time of the camera should be multiples of the DMD’s exposure time to avoid demodulation artifacts.^26^ However, despite matching our camera’s exposure time to the DMD’s exposure time (i.e., 10 ms), we did not notice significant demodulation artifacts after performing phantom calibration. We achieved this by ensuring that the trigger signal from the DMD to the camera was sent 11  μs before the pattern display to match the minimum 11  μs trigger delay time of the camera used in the current build. And as noted above, the fast frame rate at high pixel resolution may result in dropped frames during acquisition. This could be due to the bottleneck of the writing speeds of the internal SSDs. A potential solution would be saving LSI and SFDI data into separate SSDs to maximize the writing speed.

The flow phantom validation experiment showed that the system is sensitive up to 4  mm/s of directed flow speed for 1% intralipid solution. In live mouse brains, researchers have used fluorescence microscopy to measure absolute flow speed on a vascular level, where the speed of moving red blood cells ranged from 0 to 1  mm/s in capillaries, 1 to 10  mm/s in venules, and 5 to 20  mm/s in arterioles.^45^ The dynamic range of our system presents a limitation in quantitative measurements of venules and arterioles. Thus, we recommend choosing the median value of a large ROI during ROI selection and processing, such as the chosen semicircles as shown above, as the quantitative factor. This median value typically represents the parenchymal regions of the brain, where capillaries dominate.

Also, the described method of deriving $\mu_{a}$ and ${\mu_{s}}^{\prime }$ at the LSI wavelength from values obtained using SFDI can be prone to errors. This would then affect the deduced $D_{b}$ values. One solution to this problem is to perform LSI and SFDI sequentially, where LSI frames are interlaced with SFDI frames. However, this method would significantly increase the time required for the acquisition or reduce the exposure time to accommodate for a faster acquisition speed. Decreasing exposure time can increase the detectable noise levels and also affect the spatial speckle contrast measurements when the exposure time starts to approximate the correlation time (i.e., the flow would appear more static at a faster exposure time). Additionally, the sequential triggering of SFDI frames limits the SFDI frame rate to one for every 18 LSI frames. This leads to a temporal mismatch in the correction method, where 18 LSI frames, which can span the pulsatile signal, have the same optical properties. This limitation poses a potential explanation for the lack of temporal dissimilarity between $D_{b}$ and SFI, as shown in Fig. 9(b) above. Furthermore, the mismatched wavelengths raised the question of whether the two modalities probe similar tissue volumes. Here, we calculated the estimated effective depth penetration of the SFDI system using the derived optical properties at 633 nm for the 13 mice measured at resting state. The depth penetration ranges from 0.5 to ∼2  mm depending on the spatial frequencies (Table S5 in the Supplementary Material). It is acknowledged that the penetration depth for SFDI falls within this specific range. This range overlaps with the typical penetration depth of an LSI measurement.^46^ However, the exact probing volumes remain dissimilar. To address these concerns, there have recently been reports of utilizing speckle patterns as a function of spatial frequency to extract optical properties.^47^^–^^49^ However, to the best of our knowledge, a method to decouple the effect of blood flow from optical properties on speckle contrast measurements has not been reported.

### 7.2 Limitations of the Homogeneous, Semi-Infinite Tissue Assumption

A limitation of the current processing method is the employment of a semi-infinite homogeneous model for SFDI data. Furthermore, the solution from Mazhar et al. was derived for the same tissue geometry.^15^ Thus, the current correction scheme does not include the effects of volumetric heterogeneities such as the skull on the measurements. To improve the accuracy of QHI measurements of CBF, the skull should be modeled as a scattering layer with minimal absorption and a fixed $D_{b}$ value that resembles that of a static phantom.

Furthermore, it should be noted that the equations used in this publication were derived from dynamic light scattering theory with an assumption that the photons encounter multiple scattering events before being detected. This is typically true for the parenchymal region where small capillaries act as the main source of dynamics, resulting in a more diffusive behavior typically observed from multiple and unordered scattered photons.^50^ However, it was also shown that larger vessels follow a more single and ordered scattered regime. Finally, the authors also noted a mixed dynamic (i.e., multiply and ordered scattering, and single and unordered scattering) in the immediate regions surrounding these vessels. Thus, our assumption of multiple and unorder scattering potentially results in the mischaracterization of the true dynamics of the larger vessels and their surrounding regions. Nevertheless, this assumption should be appropriate for the parenchymal region, where capillaries dominate.

### 7.3 Understanding the Diffusion Coefficient and its Relevance in CBF Measurements

The diffusion coefficient $D_{b}$ is used to describe the mean squared displacement of the moving particles using the Brownian diffusion model. In diffuse correlation spectroscopy, a fiber-based analog of LSI, $D_{b}$ has been used to quantify CBF with assumed optical properties.^51^^–^^53^ Researchers have found that this Brownian model better fits the electric field autocorrelation curves compared to a random flow model when measuring the movement of red blood cells inside tissues. A potential explanation is that, in tissue, red blood cells do not simply move from one position to another but also experience shear stresses, causing them to “roll and tumble” in the vasculature.^34^ This is particularly true for the parenchymal region of the brain, where the imaging system is mainly probing small capillaries. From a macroscopic point of view, this highly randomized motion exhibits behaviors similar to Brownian motion. However, the typical values of $D_{b}$ for blood flow are magnitudes higher than that of pure thermally induced Brownian motion. As for large vessels, as discussed above, a more rigorous model should be utilized to correctly account for the more single-directional motion of the red blood cells.

The unit of $D_{b}$ is area over time (e.g., ${\mathrm{mm}}^{2}/s$). This unit differs from the clinically used units of CBF such as perfusion rate per tissue volume per time (e.g., mL/g/min). However, these clinical measurements are often obtained using a volumetric scanner, such as MRI or PET. This is different from an *en-face* widefield optical imaging measurement, such as LSI. While LSI can probe CBF at deeper brain regions, this information is usually integrated into the superficial cortical measurements. Hence, each LSI pixel represents mainly the motion detected at the surface and partial subsurface contributions. Both contribute to the blurriness of the speckle pattern at the analyzed pixel. Thus, the unit of $D_{b}$ (i.e., area per time) is appropriate to measure such dynamics. In fact, it has been shown that LSI measurements of directed motions do not measure volumetric flow directly but a combination of the channel size and the speed of the particles (i.e., mostly single-directional area flux).^54^ However, the work in that study specifically investigated the flow dynamics of large vessels, ranging from 20 to 100  μm for *in-vivo* and 65 to 175  μm for *in-vitro* demonstrations and is not directly applicable to measurements of the parenchymal regions as performed here. Future work is warranted to fully understand which model works best for each selected vascular region of the cortex.

Finally, we are interested in leveraging $D_{b}$ as a non-unitless measurement of CBF in calculating the cerebral metabolic rate of oxygen (${\mathrm{CMRO}}_{2}$). In studies combining LSI with spectroscopic imaging, relative measurements of ${\mathrm{rCMRO}}_{2}$ can be estimated with rCBF and hemoglobin concentrations using certain assumptions.^5^^,^^13^^,^^27^ A new model utilizing $D_{b}$ as a quantitative CBF surrogate will allow for a direct calculation of ${\mathrm{CMRO}}_{2}$ instead of a relative value. However, this new method requires changes to the typical ${\mathrm{CMRO}}_{2}$ equation (i.e., Fick’s law) due to the mismatched dimensions and will be the subject of future work.

## 8 Conclusion

In this work, we reiterate that the interpretation of LSI measurements can be confounded by changes in optical properties. To enable measurement of absorption, scattering, and speckle contrast, we developed and described in detail our QHI system for wide-field, small-animal neuroimaging to achieve temporal and spatial synchronization for LSI and SFDI. We demonstrated that QHI has sufficient sensitivity and precision to enable reproducible and accurate longitudinal measurements. Our *in-vivo* data suggest that CBF is most accurately described with the combination of LSI and SFDI, which has implications for comparing CBF measurements among subjects and within a given subject over time. Future work will involve the use of QHI in longitudinal studies of CBF associated with neurodegenerative disease progression.

## Code, Data, and Materials Availability

All code, data, and materials used in this study are available by reasonable request to the corresponding author.
