# Supplementary material for: Quantitative hemodynamic imaging: a method to correct the effects of optical properties on laser speckle imaging
Source: Neurophotonics. 2023 Oct 3;10(4):045001. doi: 10.1117/1.NPh.10.4.045001 (PMC10546199; doi:10.1117/1.NPh.10.4.045001)
Supplement: Supplementary file 1 [file NPh_010_045001_SD001.pdf]

Supplemental Information

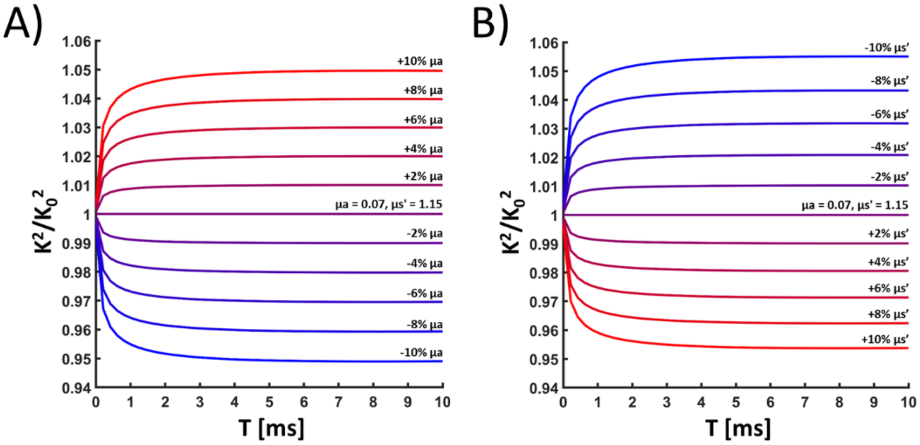

Supplemental Figure S1. Simulated changes of K^2 across a range of exposure time T due to changes of either μ_a or μ_s' using equations (9-12). All curves are normalized to a baseline K_0^2, obtained using $\mu_a = 0.07$ mm^{-1} and $\mu_s' = 1.15$ mm^{-1}. A) Varying μ_a in a range of -10% to +10% yielded a change of approximately -5% to +5% change in K^2 respectively. B) On the contrary, varying μ_s' in a range of -10% to +10% yielded a change of approximately +5% to -5% change in K^2 respectively.

		μ_a [mm^{-1}]			μ_s' [mm^{-1}]			K [a.u.]
		660 nm	780 nm	850 nm	660 nm	780 nm	850 nm	633 nm
Trial 1	Mean	0.023	0.024	0.023	1.044	0.778	0.673	0.661
	Std	2.55E-05	1.17E-05	1.85E-05	3.30E-04	2.41E-04	3.35E-04	1.74E-03
Trial 2	Mean	0.023	0.024	0.023	1.043	0.779	0.674	0.663
	Std	1.59E-05	1.33E-05	2.35E-05	2.82E-04	2.44E-04	3.85E-04	1.42E-03
Trial 3	Mean	0.023	0.024	0.023	1.043	0.778	0.674	0.662
	Std	1.23E-05	9.43E-06	1.12E-05	2.61E-04	2.13E-04	2.61E-04	1.34E-03
Trial 4	Mean	0.023	0.024	0.023	1.045	0.778	0.673	0.661
	Std	1.09E-05	1.00E-05	1.43E-05	2.44E-04	2.16E-04	2.66E-04	1.24E-03
Trial 5	Mean	0.023	0.024	0.023	1.045	0.778	0.673	0.662
	Std	1.18E-05	8.60E-06	1.21E-05	2.55E-04	1.99E-04	2.54E-04	1.28E-03
Trial 6	Mean	0.023	0.024	0.023	1.045	0.778	0.673	0.662
	Std	1.18E-05	8.60E-06	1.21E-05	2.55E-04	1.99E-04	2.54E-04	1.28E-03
Inter-trial CV [%]		0.215	0.069	0.160	0.061	0.032	0.075	0.093

Supplemental Table 1. Stability measurements over six 5-minute sessions within the same day for one static phantom of K, μ_a, and μ_s'. Mean and standard deviation measurements were reported for each session of imaging. An inter-trial coefficient of variation (CV) was calculated for all sessions of imaging. The results showed minimal drift in the measured parameters for an imaging day.

		Phantom 1			Phantom 2			Phantom 3			Phantom 4		
		660 nm	780 nm	850 nm	660 nm	780 nm	850 nm	660 nm	780 nm	850 nm	660 nm	780 nm	850 nm
μ_a [mm^{-1}]	Day 0	0.022	0.022	0.022	0.059	0.058	0.056	0.016	0.018	0.017	0.046	0.049	0.048
	Day 1	0.023	0.024	0.023	0.059	0.058	0.057	0.017	0.020	0.019	0.046	0.050	0.049
	Day 2	0.024	0.024	0.023	0.060	0.059	0.058	0.018	0.020	0.019	0.048	0.051	0.051
	Day 3	0.024	0.024	0.023	0.061	0.059	0.058	0.018	0.020	0.019	0.047	0.050	0.049
	Day 4	0.023	0.023	0.023	0.059	0.058	0.057	0.017	0.019	0.018	0.046	0.049	0.048
	CV [%]	3.8	3.1	3.0	1.6	1.5	1.5	4.3	3.7	3.8	2.0	1.9	1.9

Supplemental Table 2. Longitudinal stability measurements over five days for four static phantoms of μ_a. Measurements were reported for each wavelength on each day of imaging for each phantom. A coefficient of variation (CV) was calculated for all five days of imaging. Measured μ_a values agreed with the composition of the silicone phantoms. The results also showed acceptable stability over time (CV < 5%).

		Phantom 1			Phantom 2			Phantom 3			Phantom 4		
		660 nm	780 nm	850 nm	660 nm	780 nm	850 nm	660 nm	780 nm	850 nm	660 nm	780 nm	850 nm
μ_s' [mm^{-1}]	Day 0	1.06	0.78	0.68	1.06	0.81	0.71	2.04	1.63	1.48	1.92	1.56	1.42
	Day 1	1.05	0.79	0.68	1.07	0.82	0.71	2.04	1.64	1.49	1.96	1.58	1.43
	Day 2	1.03	0.78	0.67	1.05	0.81	0.70	2.02	1.63	1.48	1.93	1.57	1.42
	Day 3	1.02	0.78	0.67	1.02	0.80	0.70	2.01	1.63	1.49	1.92	1.57	1.42
	Day 4	1.06	0.79	0.68	1.07	0.82	0.71	2.06	1.65	1.50	2.00	1.59	1.44
	CV [%]	1.6	0.8	0.8	2.1	0.9	0.8	0.9	0.5	0.4	1.6	0.8	0.7

Supplemental Table 3. Longitudinal stability measurements over five days for four static phantoms of μ_s'. Measurements were reported for each wavelength on each day of imaging for each phantom. A coefficient of variation (CV) was calculated for all five days of imaging. Measured μ_s' values agreed with the composition of the silicone phantoms. The results also showed acceptable stability over time (CV < 2%).

		Phantom 1	Phantom 2	Phantom 3	Phantom 4
K [a.u.]	Day 0	0.65	0.68	0.56	0.64
	Day 1	0.64	0.65	0.58	0.64
	Day 2	0.64	0.69	0.59	0.65
	Day 3	0.65	0.71	0.60	0.65
	Day 4	0.64	0.70	0.60	0.66
	CV [%]	1.0	3.0	2.5	1.2

Supplemental Table 4. Longitudinal stability measurements over five days for four static phantoms of K. Measurements were reported for each day of imaging for each phantom. A coefficient of variation (CV) was calculated for all five days of imaging. Measured K values agreed with the composition of the silicone phantoms and of the proposed model from Mazhar et al. The results also showed acceptable stability over time (CV ≤ 3%).

Animal ID	μ_a [mm^{-1}]	μ_s' [mm^{-1}]	Depth penetration at 0 mm^{-1} [mm]	Depth penetration at 0.3 mm^{-1} [mm]
**1**	**0.052**	**1.26**	**2.20**	**0.52**
**2**	**0.078**	**1.15**	**1.87**	**0.51**
**3**	**0.039**	**1.37**	**2.46**	**0.52**
**4**	**0.076**	**1.26**	**1.81**	**0.51**
**5**	**0.066**	**1.40**	**1.85**	**0.51**
**6**	**0.052**	**1.25**	**2.22**	**0.52**
**7**	**0.061**	**1.17**	**2.10**	**0.51**
**8**	**0.064**	**1.09**	**2.13**	**0.51**
**9**	**0.068**	**1.25**	**1.94**	**0.51**
**10**	**0.058**	**1.22**	**2.12**	**0.51**
**11**	**0.055**	**1.15**	**2.24**	**0.52**
**12**	**0.050**	**1.18**	**2.33**	**0.52**
**13**	**0.050**	**1.20**	**2.32**	**0.52**

Supplemental Table 5. SFDI-derived optical properties at 633 nm and the estimated effective depth penetration at planar (0 mm^{-1}) and at the high spatial frequency (0.3 mm^{-1}) from 13 mice during resting-state measurements. The measurements were calculated using the equation $\delta'_{eff} = \frac{1}{\sqrt{3\mu_a(\mu_a+\mu_s)+f_x{}^2}}$. The true penetration depth of SFDI is between the two values. These values overlaps with the typical penetration depth of LSI measurements, which can range from 200 µm to 800 µm depending on the optical properties of the probed tissue.
